# Nanoporous Carbide-Derived Carbon Material-Based Linear Actuators

**DOI:** 10.3390/ma3010009

**Published:** 2009-12-24

**Authors:** Janno Torop, Mati Arulepp, Jaan Leis, Andres Punning, Urmas Johanson, Viljar Palmre, Alvo Aabloo

**Affiliations:** 1 IMS Lab, Institute of Technology, University of Tartu, Nooruse1, 50411 Tartu, Estonia; E-Mail: janno.torop@ut.ee (J.T.); 2 Carbon Nanotech Ltd., Riia 185, 51014 Tartu, Estonia; 3 Institute of Chemistry, University of Tartu, Ravila 14a, 50411, Tartu, Estonia

**Keywords:** linear actuator, porous carbon, ionic liquid, carbide-derived carbon, Electroactive Polymer (EAP) actuator

## Abstract

Devices using electroactive polymer-supported carbon material can be exploited as alternatives to conventional electromechanical actuators in applications where electromechanical actuators have some serious deficiencies. One of the numerous examples is precise microactuators. In this paper, we show for first time the dilatometric effect in nanocomposite material actuators containing carbide-derived carbon (CDC) and polytetrafluoroetylene polymer (PTFE). Transducers based on high surface area carbide-derived carbon electrode materials are suitable for short range displacement applications, because of the proportional actuation response to the charge inserted, and high Coulombic efficiency due to the EDL capacitance. The material is capable of developing stresses in the range of tens of N cm^-2^. The area of an actuator can be dozens of cm^2^, which means that forces above 100 N are achievable. The actuation mechanism is based on the interactions between the high-surface carbon and the ions of the electrolyte. Electrochemical evaluations of the four different actuators with linear (longitudinal) action response are described. The actuator electrodes were made from two types of nanoporous TiC-derived carbons with surface area (S_A_) of 1150 m^2^ g^-1^ and 1470 m^2^ g^-1^, respectively. Two kinds of electrolytes were used in actuators: 1.0 M tetraethylammonium tetrafluoroborate (TEABF_4_) solution in propylene carbonate and pure ionic liquid 1-ethyl-3-methylimidazolium trifluoromethanesulfonate (EMITf). It was found that CDC based actuators exhibit a linear movement of about 1% in the voltage range of 0.8 V to 3.0 V at DC. The actuators with EMITf electrolyte had about 70% larger movement compared to the specimen with TEABF_4_ electrolyte.

## 1. Introduction

Electroactive materials can respond to electrical stimulus with a significant shape or size change. Polymer-composite electroactive materials are the object of interest due to their small dimensions and the possibility to make displacement in any direction. These materials can be cut (almost) into any shape and form, and some of these materials, for example, like ionic polymer metal composites (IPMC), may work at relatively low voltages (1–5 V) [1,2,3,4]. These properties make them valuable as mechanical actuators in applications where conventional actuators have natural limitations. One of the promising areas of application is biomedical devices. EAP actuators are soft, easy to miniaturize and some of them can operate at low voltages.

However, there are some issues with these actuator materials that are not completely solved. The main problems are the short lifetime, the complexity of the manufacturing process, insufficient reproducibility of properties of formed actuators, and the insufficient control and guidance of the actuator movement. It has been reported that the movement depends mainly on the charge used and not on the applied voltage. High-speed actuators with relatively high power are still a challenge [2,5].

Several types of carbon based electroactive materials are widely known [5]. Carbon nanotubes (CNTs), fullerenes and graphene-like structures are some of the attractive candidates for actuators. These materials have uniquely high mechanical actuation force properties, but low strain [6]. Carbon based actuators predicate on two main actuation mechanisms. The first principle is based on the electronic conductivity of carbon material. Actuators of such type need high electrical potential (field) for actuation [7]. Actuation occurs due to carbon-carbon interaction change due to high electrical field. Another principle is diffusion of ions and ion pairs induced by applied low potential. These transducers usually combine carbon materials with polymer matrix and some ionic conducting media. They seem to have much more possible applications in the near future [8,9].

Che Guangli *et al*. made the first example of an actuator based on a nanostructured carbon material. The actuator consisted of branched carbon nanotubules embedded within the pores of a microporous alumina template membrane and graphitic carbon used as the electroactive material. Electrochemical Li^+^ intercalation caused this nanotubule-containing membrane to flex, and electrochemical de-intercalation causes the membrane to relax to its original position [10].

CNTs have been one of the most intensively studied carbon materials [1]. The first actuator made of CNTs was a macro scale sheet of nanotubes termed bucky–gel paper [12,13,14,15]. This actuator produced strain due to the change in dimension of the nanotubes in the covalently bonded direction caused by an applied electric potential. The created excess charge is compensated at the nanotubes—electrolyte interface by the electrolyte ions forming a double layer. The charge induces ion movement, which leads to a change in the dimension of the nanotube paper causing the assembly to bend. Soon after the bucky paper actuator demonstrated actuation of single-walled carbon nanotubes (SWNTs), in which the SWNT were suspended over a trench, and the edges fixed at the surface with a metal layer. The charging leads to a rearrangement of the electronic structure of the nanotubes, and to Coulombic forces, both of which result in dimensional changes [14].

Besides CNTs, several microporous- and graphitic carbons were studied by means of dilatometry by Campana and Hahn *et al.* [18]. The graphitic carbon exhibited highest volume change (10–15%), whereas the expansion was observed at voltages between −2.0 and +2.5 V (ΔU = 4.5 V). The microporous carbons had the expansion about ~5% at voltages below -1.0 V and above +1.0 V (ΔU = 2.0 V) [18]. The *in situ* Raman spectroscopy technique was applied to study ion intercalation behavior into the carbon body [19].

Behind several traditional nanostructured carbons like CNTs, fullerenes and graphenes, high surface area amorphous carbon is also an interesting candidate for actuators. The actuation phenomenon of porous carbon was discovered while searching for appropriate materials for new alternative energy storage devices [21].

**Figure 1:**
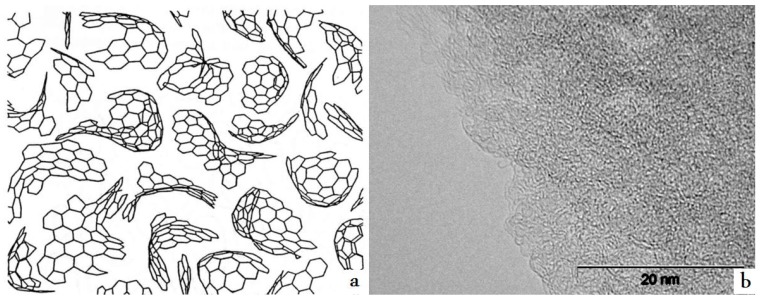
A structure (a) [7] and High Resolution Transmission Electron Microscope (HRTEM) picture (b) of amorphous microporous carbon.

Microporous amorphous carbons have non-uniform distribution of the curved graphene flakes as shown in Figure 1. Carbon atoms have hybridization of sp^2^, but graphene particles form 3D network. Due to non-uniform structure, it is quite difficult to describe porous carbon, because HRTEM images only show a very small area of samples [22].

So called template carbons based on metal carbides are of interest in energy storage devices beside widespread activated carbons commonly produced by thermal decomposition of organic precursor materials and successive water vapor activation, because the pore size is tunable due to the synthesis parameters and by modifying the template. One representative of such template carbons is carbide-derived carbon (CDC), which is obtained by extracting the non-carbon atoms from the carbide lattice. CDCs have been investigated as promising candidates for electric double layer capacitor (EDLC) electrode materials, for low molecular weight gas adsorbents (CH_4_, H_2_, *etc.*) and for electron emission sources [23,24]. These materials usually possess high surface area (1,000–2,000 m^2^ g^-1^), controlled micro-pore volumes and pore size distributions. The advanced CDC materials have high electric double layer (EDL) capacitance (130 F g^-1^ and 90 F cm^-3^ in nonaq.), high electric energy conversion efficiency (up to 98%), very good material stability and the chemical inertness. Supercapacitors based on CDCs have shown electrode lifetimes up to 10^6^ cycles [25].

The expansion of the carbonaceous electrodes is generally undesired, and is a crucial factor for the lifetime of energy storage devices. Hahn *et al*. have performed dilatometric studies on porous and graphitic carbon electrodes and observed significant reversible volumetric change of the electrodes during the charging-discharging. This confirms the electroactive properties of porous carbon [18,19,20].

The volume changes of the supercapacitor materials during the charging process were previously observed by Hahn *et al.* [17,18]. In principle, the design of the ionic polymer actuator described in this paper is comparable to supercapacitors. Thereby, relying on association by similarity, the electroactive mechanism of nanoporous carbon electrodes is related to electric double-layer formation during charging of the material, and the dimensional change of the actuator is the consequence of charge injection in carbon nanopores during the electric double-layer charging. It is also reported that particular gas evolution (Faradic process) on the micro-pores could be a reason of the carbon actuation process, but using TEABF_4_ salt in propylene carbonate solution and ionic liquid EMITf the electrochemical reactions are marginal in the voltage range of 0 to 3.0 V [27,28,29].

In this paper, we show for first time the dilatometric effect in actuators containing the CDC materials. Transducers based on a high surface area carbide-derived carbon electrode materials are suitable for short range displacement applications, because of proportional actuation response to the charge inserted and high Coulumbic efficiency due to the EDL capacitance. The films/sheets made from the material change their thickness, so it is possible to fabricate a device with linear actuation properties.

## 2. Preparation of Actuators

### 2.1. Synthesis of Carbide-Derived Carbon

Titanium carbide loaded into the horizontal quartz reactor was reacted with a flow of chlorine gas (99.999% assay) at reaction temperatures 600 °C or 800 °C until all the carbide was converted into carbon. The flow rate of chlorine gas was set so that the optimum mass transfer of gaseous reactants and products occurred, which depended on reaction volume, and the reaction temperature. The mass balance of the titanium carbide chlorination reaction is expressed by Equation 1, which describes the corresponding chemical reaction:

(1) $$TiC+2Cl_{2}\to C+TiCl_{4}↑$$

The by-product, TiCl_4_, was evacuated from reactor by the stream of the excess chlorine. During the heat-up and cooling phase, the reactor was flushed with a slow stream of argon. Resulting carbon powders were finally purified in the flow of hydrogen at 800 °C. The sample chlorinated at 800 °C was additionally oxidized according to the method of the high-temperature treatment of the carbon previously saturated with the trapped water in the nanopores [30].

### 2.2. Preparation of the Electrodes

CDC powder (1 g) was mixed with a small amount of ethanol and 10% (by wt.) of PTFE (as a 60% suspension in water) to form a slurry, which thereafter was gently pressed until a wet material was formed. After evaporation of ethanol, the material was impregnated with heptane, shaped to a cylinder and rolled in the axial direction of the cylinder. The latter procedure was repeated until elastic properties appeared in the carbon sheet. Finally, the heptane was removed at ~110 °C, the carbon sheet was rolled stepwise down to the desired thickness, dried under vacuum at 170 °C and coated from one side with a thin (~2 μm) aluminum layer, using Plasma Activated Physical Vapor Deposition. Electrode discs of desired size were cut from these sheets.

### 2.3. Assembling of the Actuators

**Figure 2:**
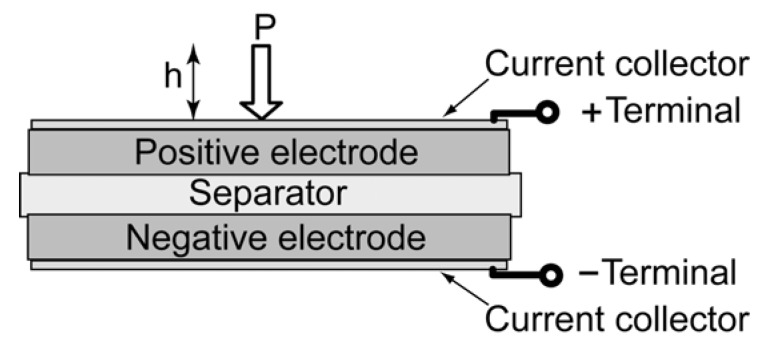
Schematic of the linear actuators.

Linear actuators 1–4 (see Table 1) were assembled from pairs of carbon electrode discs (∅ 1.7 cm), separated with an ion-permeable separator paper from Kodoshi Nippon (cf. Figure 2). Each actuator consisted of five electrode pairs with a total geometric surface area of 11.35 cm^2^. The electrode assembly was placed in a sealed aluminum container. Additional mechanical pressure (p = 48 N cm^-2^) was applied by a spring through a special connecting rod. This rod was further used for measuring the linear movement of actuator by a laser beam.

The actuator cells were held under vacuum at 120 °C for 24 h before being impregnated with the electrolyte. The electrolytes used in this study were 1.0 M tetraethylammonium tetrafluoroborate in propylene carbonate (“Honeywell Digirena ®” TEABF_4_/PC, H_2_O < 0.003%) and the ionic liquid 1-ethyl-3-methylimidazolium trifluoromethane-sulfonate (EMITf, Fluka).

**Table 1:** The actuators used in this study.

Actuator #	Notation of actuator	Notation of CDC material	Electrolyte
**1**	TEA600	TiC-600	1.0 M TEABF_4_/PC
**2**	TEA800	TiC-800	1.0 M TEABF_4_/PC
**3**	EMI600	TiC-600	EMITf
**4**	EMI800	TiC-800	EMITf

Before evaluation, the actuator cells were kept at 60 °C for 48 h, to saturate the compact nanoporous electrode body with the electrolyte. Thereafter, continuous galvanostatic cycling between 2.7 V and 0.05 V with the current *I* = 100 mA was carried out, prior to performing further studies. The purpose of preliminary galvanostatic cycling is to distribute the electrolyte ions and decompose the traces of H_2_O as well as to inactivate other possible impurities. Usually five cycles were needed to establish a stable actuation of the electrochemical system.

### 2.4. Evaluation of Carbon Materials

The low-temperature nitrogen sorption experiments were performed at the boiling temperature of nitrogen (77 K). The specific surface area (S_A_) was calculated according to the BET theory [31] up to the nitrogen relative pressure (P/P_0_) of 0.2. The total volume of pores (V_p_) was calculated at relative pressure (P/P_0_) of 0.95 and the volume of the micropore (V_μ_) from t-plot, using Harkins-Jura thickness values between 5 Å and 90 Å calculated according to Equation 2:

(2) $$t=\sqrt{\frac{13.99}{0.034-log\frac{p}{{\text{P}}_{0}}}}$$

Average pore size (APS) is calculated according to the Equation 3:

(3) $$\text{APS}=\frac{2{\text{V}}_{\text{p}}}{{\text{S}}_{\text{A}}}$$

**Table 2:** Some physical parameters of TiC derived carbons.

Notation of CDC material	T_Chlorine_ [°C]	S_A_ [m^2^ g^-1^]	Vp [cm^3^ g^-1^]	V_μ_ [cm^3^ g^-1^]	APS [Å]
TiC-600	600	1150	0.53	0.49	9.3
TiC-800	800	1470	0.71	0.59	9.7

## 3. Electrochemical Properties

### 3.1. Experimental Setup

Electrochemical characterization of the actuators was performed in a sealed electrochemical cell at room temperature, by using the Solatron potentiostat 1287 with FRA analyzer. Cyclic voltammetry (CV), constant current (CC), constant potential (CP) and electrochemical impedance spectroscopy (EIS) experiments were carried out. The CV was performed at different voltage sweep rates: 2, 5, 10, and 50 mV s^-1^. The region of working voltage was varied in the interval of: 0–2.5 V, 0–2.7 V and 0–3 V. The galvanostatic measurements were performed in the current range of 10–1000 mA at voltages from 0.05 V to 3 V. The impedance spectra were recorded at fixed DC potentials: 1.5 V, 2.5 V, and 2.7 V, using an AC signal of 5 mV.

### 3.2. Electrochemical Measurement Results

**Figure 3:**
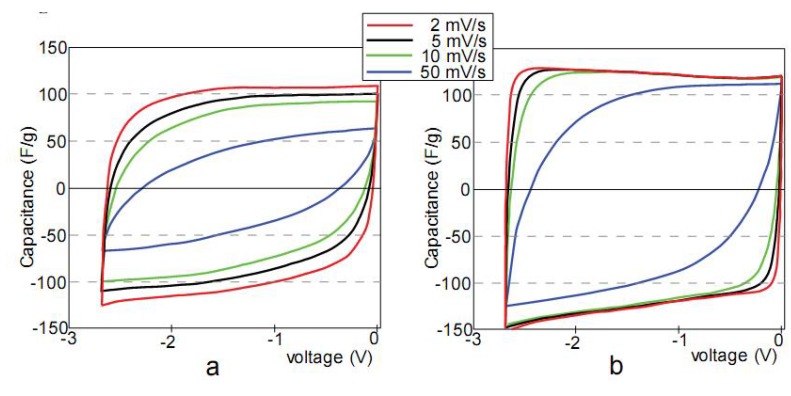
Capacitance *vs*. voltage of the actuators EMI600 (a) and EMI800 (b) at different voltage sweep rates.

The charge-discharge capacitance calculated from the cyclic voltammetry plots of actuators is shown in Figure 3a and b. Note that the specific capacitance is expressed per dry weight of carbon (F g^-1^).

**Figure 4:**
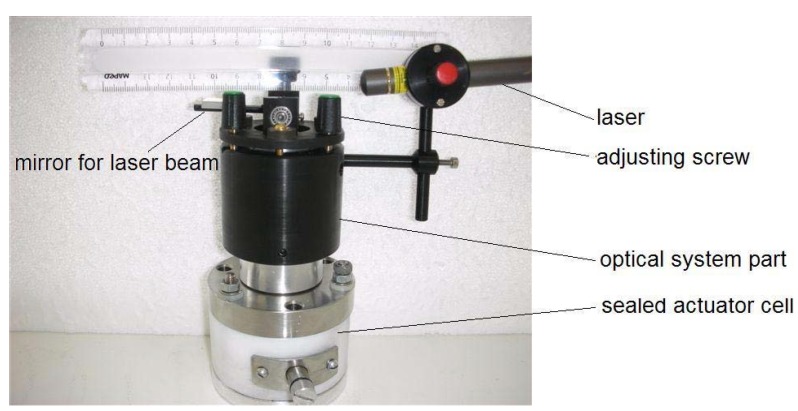
The linear actuator test cell.

The cyclic voltammetry analyses of the actuators show a non-faradic charging behavior, and the electrical double layer capacitance is predominant in the voltage range of 0 to 2.7 V. Generally, it is known that the specific capacitance may depend on the voltage sweep rate. In our study, the main difference between the linear actuators EMI600 and EMI800 is the carbonaceous material used in their manufacture. The actuator EMI600 was made from nanoporous TiC-600 carbon with an average pore size (APS) of = 9.3 Å, whereas carbon in the actuator EMI800 has a slightly increased APS of 9.7 Å. In Figure 4a and b, it is seen that in case of EMI600 the potential sweep rate has a large influence on the capacitance. However, the specific capacitance of EMI800 is almost independent of voltage sweep rate. The observed differences in specific capacitance can be explained by different nanostructure and pore distribution of investigated carbon materials. TiC-800 has a sparse media with slightly higher pore volume compared to TiC-600. Hence, a sparse microstructure of TiC-800 provides higher mobility of electrolyte ions and dependencies to voltage sweep rate will become obvious at higher values. Similar behaviors were observed for TEA600 and TEA800, which differ from the EMI600 and EMI800 by the electrolyte used (1.0 M TEABF_4_/PC *vs*. EMITf).

The EIS measurements were carried out at an AC signal of 5 mV and fixed DC potentials of 2.5 V and 1.5 V in frequency range of 1 MHz to 5 mHz. The capacitance (C_S_) was calculated at the frequency of 10 mHz and resistance of the actuators (R_S_) at 100 Hz using the model of a series RC-circuit. The phase angle (Θ) for the ideal capacitors is –90°. In current study, the measured phase angle values of the actuators were in the range of –55° to –72°. The EMI600 sample has higher resistance than EMI800, and it has also lower capacitance than EMI800. This phenomenon can be explained by the fact that both the EMI600 and EMI800 devices used the same EMITf electrolyte which has an unsymmetrical cation with an ionic radius of 7.6 × 4.3 Å, but an almost symmetric anion of ~4 Å. Apparently, part of the nanopores in EMI600 are not suitable for adsorbing the cations due to the steric restrictions, which results in the decreased capacitance and the increased internal resistance. This fact is partly proven by the data of EMI800, which uses the same electrolyte, but has slightly larger nanopores in the carbon. The EMI800 has highest capacitance and also low internal resistance, which indicates on the best fit between the porous structure and the size of electrolyte ions. The calculated data from the EIS measurements is presented in Table 3.

**Table 3:** The series capacitance (C_s_), series resistance (R_s_) and phase angle (Θ) obtained from EIS spectra.

Notation of actuator	C_s_ [F g^-1^]	R_s_ [Ω cm^2^]	Θ [ °]
TEA600	64	3.9	-71
TEA800	112	3.6	-72
EMI600	75	7.3	-55
EMI800	121	4.6	-71

## 4. Electromechanical Characterization

### 4.1. Electromechanical Measurement Modes

The extent of actuation of the actuators in time-domain was detected using a custom-made optical system. The measurements were performed in two modes:

1. the constant current charge-discharge process (CC mode);
2. the current of the actuator is limited and the relatively short period of constant current is followed by a prolonged period of constant potential (CCCP mode).

### 4.2. Experimental Setup

**Figure 5:**
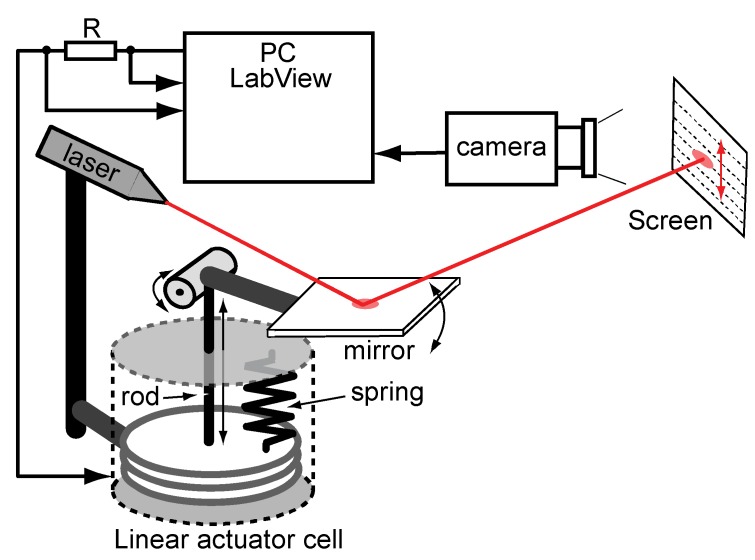
Experimental setup.

The test cell of the linear actuator is depicted in Figure 4. It consists of two fundamental parts: the electrochemical actuator and the optical detecting mechanism. The actuator is assembled in a sealed cell. The cell incorporates a spiral spring, compressing the actuator by the force of 100 N. The optical system is mounted on the top of the cell. The mechanical coupling between the actuator and the optical system is implemented by a shifting vertical rod. The movement of the rod is transferred to the moving mirror using a steel eccentric and a lever. This mechanics magnifies the scale of movement of the actuator by 25 times. Further magnification of the scale is performed by a laser beam, reflecting from the mirror and targeted to a distant screen, as depicted in Figure 5.

The described optical system is able to magnify the scale of movement of the linear actuator by thousands of times, depending solely on the distance of the screen. In the course of the experiments, the distance to the screen was 6.25 m and the expansion of the actuator by 1 μm was detectable as a displacement of the spot of the laser beam on the screen by 1 cm. The displacement of the spot on the screen was recorded by a Firewire camera and the motion was thereafter digitalized using LabView software. Simultaneously, the electrical parameters—voltage and electric current—were recorded using a National Instruments PCI-6034 DAQ card and LabView software. The electric current was measured as a voltage drop over the low ohmic resistance R.

### 4.3. CC Mode Experiments

**Figure 6:**
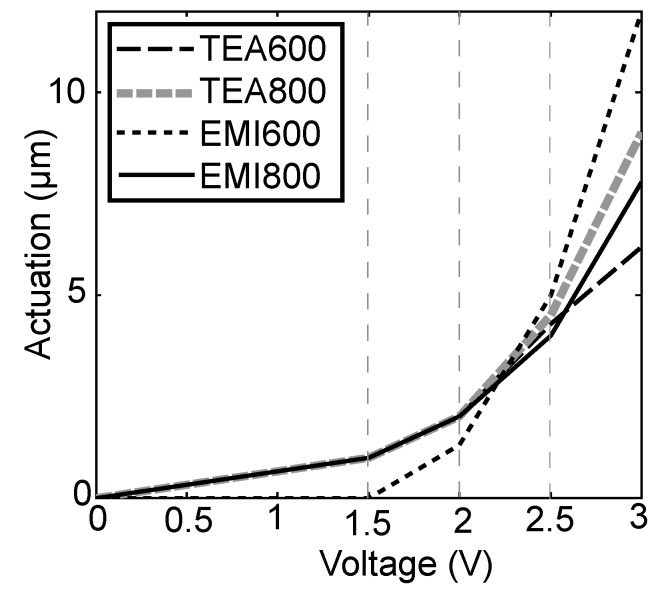
The actuation of the actuators at different voltages obtained at charging current *I_C_* = 10 mA.

In this mode, the actuation amplitude was estimated using the constant current charge-discharge process. The amperage of the constant current *I_C_* was varied in the range of 10 to 400 mA. The actuation amplitude was recorded simultaneously with charging or discharging current characteristics of the actuator. The potential of the electrodes gained some certain predefined values, e.g., 1.5, 2.0, 2.5 and at 3.0 V. The results of the measurements are given in Figure 6.

The graph reveals that the four actuators function almost identically—the difference between the amplitudes of actuation is less than 20%—for voltages up to 2.5 V. At higher voltages the dissimilarity increases. At 3.0 V the difference between the actuation of TEA600 and EMI600 is almost twofold.

In CC mode, the electrical energy *E* is derived from the voltage diagram according to Equation 4.

(4) $$E=\int_{t_{1}}^{t_{2}}U\left(t\right)dt$$

It is possible to evaluate the energy loss *E_loss_ = E_out_ − E_in_* of the actuators during a single working cycle by defining the electrical input energy (*E_in_*) of the actuator as the energy of its charging current and defining the output energy (*E_out_*) as the energy of discharging current. The calculated energies at different values of charge-discharge currents are given in Table 4. The loss of energy is contingent on charging, relocating the ions, and probably on the microscopic dimensional changes. Another possible reason of energy loss can be the ongoing faradic process. However, the impedance spectroscopy of the actuators did not reveal any of it.

**Table 4:** Electrical energies (J) and maximum actuation of the linear actuators (μm) at different amperages of the charge-discharge current *(ΔU* = 3.0 V).

*I_C_*	70 mA				300 mA			
	*E_in_* (J)	*E_out_* (J)	*E_loss_* (J)	*actuation* (μm)	*E_in_* (J)	*E_out_* (J)	*E_loss_* (J)	*actuation* (μm)
TEA600	15	11.9	3.1	4.0	12.2	6.6	5.6	2.4
TEA800	25	16	9	8.0	18.9	11.3	7.6	5.0
EMI600	18.9	11.5	7.4	7.2	8.3	2.8	5.5	1.5
EMI800	25	19.8	5.2	6.0	20	10.8	9.2	3.0

Table 4 shows that there exists a correlation between the energy loss and the amplitude of actuation of the actuators at z charging current of 70 mA. TEA600 has lowest actuation value with minimal energy loss during charging-discharging cycle. The TEA800 actuator surpassed the other studied actuators in actuation rate at 70 mA. TEA800 also possessed the highest energy loss value when measurements were performed at 70 mA. This indicates that a part of electrical energy is transferred into mechanical energy and loss of energy during charging cycle is proportional to the displacement generated by the actuators. The charge injection mechanism for the actuation of carbon is non-faradaic, whereupon the charging of the actuator of capacitive nature causes the relatively large changes in the covalent bond lengths due to the structure of the band and the Coulombic effects. The actuation is caused by the microscopic dimensional changes of the carbon-polymer electrodes.

At the charging current of 300 mA higher actuations were exhibited by the TEA800 and EMI800. In CC mode, the actuation produced by actuators with the nanoporous carbon TiC-600 is weaker than of those containing TiC-800. The correlation clearly appears at high charging current values. Polymer-supported carbon electrode materials have internal resistance, retarding the propagation of electric field in electrode sheets. At relatively high charging current values, the actuation of porous carbon material is limited by diffusion, which excludes the quick actuation response. The amount of confined ions in nanopores can be enlarged by increasing the diffusion rate. Therefore TiC-800 carbon material is preferred to give quick actuation response, due to its slightly macroporous 3D network compared to TiC-600. Viscosity of the electrolyte cannot be neglected when aiming at quick response of an actuator. TEABF_4_/PC electrolyte has an observable advantage when used in TiC-800 actuator to generate rapid electromechanical response. In TiC-600, the effect of the low value of viscosity of TEABF_4_/PC is reduced by the fact that TiC-600 has narrower pores and TEABF_4_ will render up the PC solvent shell when entering into the nanopores [30].

### 4.4. Estimation of Actuator Efficiency

The electric energy conversion efficiency or so-called round trip efficiency (RTE) of a supercapacitor depends significantly on the current used in charging-discharging cycle. Usually establishing EDL capacitance of supercapacitors, they were charged with relatively low current densities. Actuators of this study were charged with relatively high current densities, therefore RTE of actuators is somewhat lower than similar capacitors discussed elsewhere [25]

The energy conversion efficiencies of electrical energy to mechanical energy of the actuators were calculated in two different ways (see Equations 5 and 6). Respective values are presented in Table 5.

(5) $$\text{Efficiency}\left(E_{in}\right)=\frac{F\times s}{E_{in}}\times 100\%$$

(6) $$\text{Differential Efficiency}\left(E_{loss}\right)=\frac{F\times s}{E_{in}-E_{out}}\times 100\%$$

In Equations 5 and 6, F is a force inflicted by the spring and s is the displacement of actuators during charging. E_in_ and E_out_ are input and output energies calculated from CC mode according to Equation 4. As discussed in the previous section, E_in_-E_out_ can be defined as an energy loss of the actuators. One component of energy loss is the energy generated by the actuator to compress the spring during the actuation. Other components of energy loss are related to carbon material inner resistance and thermal effects occur during charging cycle. E_in_ is the total electric energy inserted in the actuator during charging and efficiency calculated by E_in_ characterizes the situation when output energy E_out_ cannot be collected. In reality, collecting of output energy of actuators is convenient and therefore implementation of output energy increases the efficiency of actuators. Efficiencies in Table 5 are calculated from data collected by charging actuators with 70 mA and 300 mA. At a current value of 70 mA, the average differential efficiencies of the actuators are two times higher than the value calculated at 300 mA, however efficiencies calculated by E_in_ are almost comparable at both charging current values and this is particularly true for TEABF_4_/PC electrolyte. The highest overall efficiency value 0.014% was observed for actuator TEA600. At current value 70 mA TEA600 has actuation 4.0 μm, which is lowest value compared to other actuators and respective energy loss was 3.1 J (see Table 4). Thereby, the high efficiency of TEA600 is mainly provided by low energy loss value. At 300 mA charging current value the differences in actuators efficiencies calculated by E_in_ decreases compared to respective values calculated at 70 mA, because the amount of energy that can be inserted at 300 mA is significantly smaller than at 70 mA.

**Table 5:** Calculated efficiencies of the actuators at different charging current values.

*I_C_*	70 mA		300 mA	
	Efficiency by E_in_ (%)	Differential efficiency by E_loss_ (%)	Efficiency by E_in_ (%)	Differential efficiency by E_loss_ (%)
TEA600	0.0029	0.014	0.0022	0.0047
TEA800	0.0035	0.01	0.0029	0.0072
EMI600	0.0042	0.011	0.002	0.0047
EMI800	0.0026	0.013	0.0017	0.0036

### 4.5. CCCP Mode Experiments

In order to examine the dynamics and peak of the actuation of the actuators, the CCCP mode was used—after charging the actuator to a certain voltage using a constant current *I_C_*, a constant potential *V_C_* was applied for up to 5 minutes. During the period of the constant potential, the current decreases substantially and the peak of actuation appears in few minutes. In the course of different experiments, the amperage of the constant current was varied in the range of 50 to 1,000 mA.

**Figure 7:**
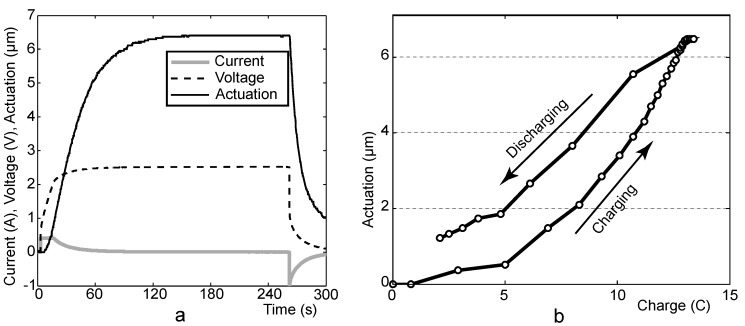
CCCP measurement of EMI800 (I_C_ = 400 mA, V_C_ = 2.5 V). Voltage, current and actuation *vs*. time (a) and actuation *vs*. charge (b).

Figure 7a depicts the diagrams of charging the actuator EMI800 in the CCCP mode. The actuation culminates and remains stable in about 120 seconds, while the voltage reaches its stable level already in 60 seconds. This diagram is very typical, similar behavior was observed for all tested actuators. The actuation-charge diagram depicted in Figure 7b indicates that there exists a nonlinear correlation between the actuation and the electric charge of the actuator.

**Figure 8:**
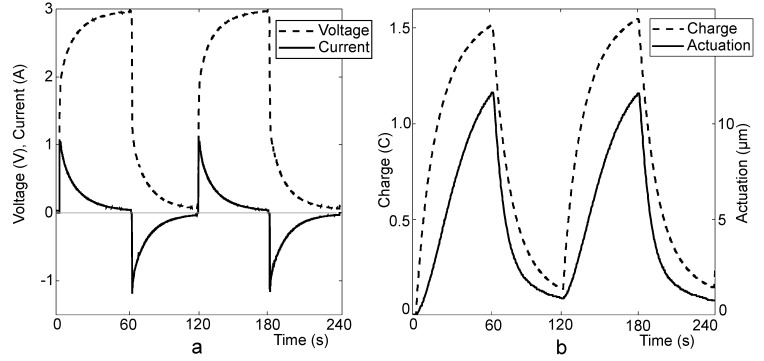
CCCP measurement of EMI800 (I_C_ = 1000 mA, V_C_ = 3 V). Voltage and current *vs*. time (a); actuation and charge *vs*. time (b).

Figure 8 depicts the series of periodic charging-discharging cycles of the actuator. As the charging current is high, the period of constant current ceases already in a few seconds. During this time interval almost no actuation is detected, since the voltage is low and the adsorption of ions is a diffusion-limited process in the porous electrodes. The current dies away slowly due to the internal resistance of the actuator. While discharging by short-circuiting, the adsorbed energy is wasted through the low ohmic discharge resistor.

The actuation throughout the simultaneous cycles is of similar characteristics. The actuation peak (12 μm) is reached in the both cycles of Figure 8. The average speed of actuation can be calculated from the data of actuation, and is 0.2 μm s^-1^. Due to the low speed of desorption of ions of the ionic liquid from the porous carbon, the process of discharging is slow and the cell does not relax to its initial thickness between the simultaneous cycles. The actuators are able to gain their initial position after only about 5 minutes shorting.

**Figure 9:**
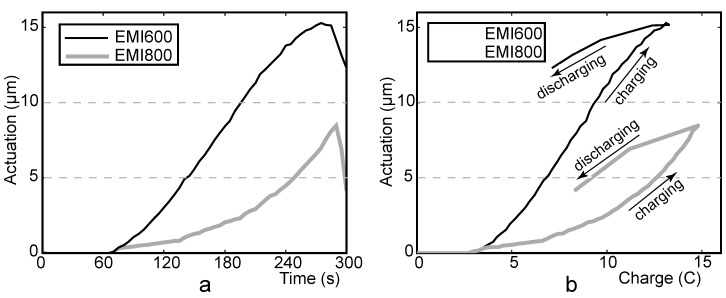
Actuation of EMI600 and EMI800 *vs*. time **(a)** and *vs*. charge **(b)** (I_C_ = 50mA; V_C_ = 3V).

Figure 9 depicts the comparison of the actuators EMI600 and EMI800. Both actuators expand slowly as the relatively low current (I_C_ = 50 mA) gives to the systems enough time to charge the double layer of carbon electrodes. The graph given in Figure 9a indicates that EMI600 is faster than EMI800. After 200 seconds of charging, the actuations of the two systems differ about four-fold and EMI600 requires less charge for the same degree of actuation. When the charge of the actuators reaches 10 C, the difference between the amplitudes of actuation of the two systems is about four-fold.

**Figure 10:**
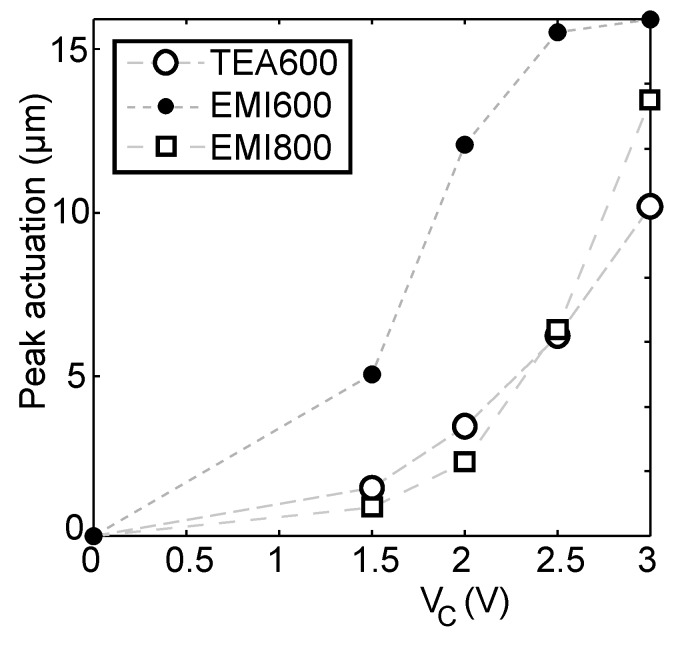
Peak actuation in the case of different constant potentials (I_C_ = 400mA).

Figure 10 represents the extreme expansion of the actuators in the case of different constant potentials of the CCCP mode. The highest actuation was generated by EMI600. It reaches its ultimate amplitude of actuation at 2.5 V already, while the actuations of TEA600 and EMI800 are lower by about half. In CCCP mode, the actuators consisting of the highly nanoporous carbon TiC-600 exhibit higher motion when compared to the carbon TiC-800 in EMITf electrolyte. The reason of their performance can be revealed by comparing the sizes of the pores of carbon and the sizes of the ions of the ionic liquid. The average nanopore size of TiC-600 is 9.3 Å—approximately close to the size of the cations of EMITf. The density of the TiC-600 carbon material is higher than that of TiC-800. 70% of the pores of the material TiC-600 are smaller than 11 Å, for the material TiC-800 the corresponding value is 50%. Electrolyte ions evoke higher dimensional changes when they enter into the nanoporous carbon media with narrower pores.

EMI^+^ and Tf^-^ appear in ionic liquid as ion pairs with much larger ionic dimensions, compared to TEA^+^ and BF_4_^¯^ in PC electrolyte. The volume changes of the actuator depend on the volume difference between cation and anion of the electrolyte used [32].

## 5. Conclusions

Novel linear electromechanical actuators based on TiC-derived carbon material were prepared and studied. The actuators consist of carbon material thin films and an electrolyte. Two different nanoporous carbons were used to prepare actuators combined with TEABF_4_ in PC and EMITf electrolytes. The method for TiC-derived carbon synthesis offers a unique control over carbon microstructure and allows specific tuned porosity of the material. Therefore, it is possible to design a structure of CDC material to best suit the electrolyte used, and to enhance the performance of the actuators. During continuous cycling, good reproducibility of actuation was observed. Displacement of the actuators was measured simultaneously to the electrochemical measurements, and from where the electric energy and mechanical energy dependencies were calculated. The mechanical energy generated by an actuator was found to be five orders of amplitude smaller than the electrical energy provided to the actuator during charging, however during discharging at least 80% of the stored electrical energy can be collected, which improves the efficiency of the actuators by almost two orders of magnitude. The advantage of described linear actuator is not only the considerably high force generated during actuation, but also the long life cycle and high capacitance due to the device being evolved from a material commonly used in supercapacitors.
